# Impact of Deoxynivalenol and Zearalenone as Single and Combined Treatment on DNA, Cell Cycle and Cell Proliferation in HepG2 Cells

**DOI:** 10.3390/ijms24044082

**Published:** 2023-02-17

**Authors:** Ana-Marija Domijan, Klara Hercog, Martina Štampar, Goran Gajski, Marko Gerić, Marijana Sokolović, Bojana Žegura

**Affiliations:** 1 Faculty of Pharmacy and Biochemistry, University of Zagreb, 10000 Zagreb, Croatia; 2 Department of Genetic Toxicology and Cancer Biology, National Institute of Biology, 1000 Ljubljana, Slovenia; 3 Mutagenesis Unit, Institute for Medical Research and Occupational Health, 10000 Zagreb, Croatia; 4 Poultry Center, Croatian Veterinary Institute, 10000 Zagreb, Croatia

**Keywords:** mycotoxins, comet assay, flow cytometry, co-exposure, food monitoring

## Abstract

The study aimed to investigate toxicity and the mechanism of toxicity of two *Fusarium* mycotoxins, deoxynivalenol (DON) and zearalenone (ZEA). DON and ZEA were applied to HepG2 cells as single compounds and in combination at low environmentally relevant concentrations. HepG2 cells were exposed to DON (0.5, 1, and 2 µM), ZEA (5, 10, and 20 µM) or their combinations (1 µM DON + 5 µM ZEA, 1 µM DON + 10 µM ZEA and 1 µM DON + 20 µM ZEA) for 24 h and cell viability, DNA damage, cell cycle and proliferation were assessed. Both mycotoxins reduced cell viability, however, combined treatment with DON and ZEA resulted in higher reduction of cell viability. DON (1 µM) induced primary DNA damage, while DON (1 µM) in combination with higher ZEA concentrations showed antagonistic effects compared to DON alone at 1 µM. DON arrested HepG2 cells in G2 phase and significantly inhibited cell proliferation, while ZEA had no significant effect on cell cycle. The combined treatment with DON and ZEA arrested cells in G2 phase to a higher extend compared to treatment with single mycotoxins. Potentiating effect observed after DON and ZEA co-exposure at environmentally relevant concentrations indicates that in risk assessment and setting governments’ regulations, mixtures of mycotoxins should be considered.

## 1. Introduction

Mycotoxins are secondary metabolites of moulds that are often found as contaminants on various commodities intended for animal and human consumption. Mycotoxins produced by *Fusarium* species, i.e., *Fusarium* mycotoxins are of particular concern since they are commonly found on maize, barley and wheat all around the world. Large-scale global survey conducted from 2008 to 2017, in which more than 74,000 samples of feed raw materials and feed in 100 countries around the world were collected, found that 88% of samples were positive for at least one mycotoxin while 64% of samples were positive for more than two mycotoxins [1]. The most frequent encountered co-contamination was with *Fusarium* mycotoxins, deoxynivalenol (DON) and zearalenone (ZEA) [1,2]. DON and ZEA co-contamination of maize as well as beer samples was found in surveys conducted in several European countries [3,4]. The analysis of human urine samples confirmed human co-exposure to these two mycotoxins [5,6], and a UK study that detected DON in 98.7% of human urine samples, clearly associated cereal intake and presence of DON in urine samples [7].

DON and ZEA have potential toxic effect to animals. In experimental animals, DON causes vomiting, decrease of weight gain, anorexia, diarrhoea, gastroenteritis and alters the immune system [8,9]. The most prominent effect of ZEA is its oestrogenic potential. In experimental animals, ZEA affects organs such as uterus, ovary, mammary glands and testes leading to malignancies of these organs [10,11]. The oestrogenic potential of ZEA is attributed to similarity of its chemical structure to 17 β-estradiol, that enables ZEA to competitively bind to oestrogen receptors and affects oestrogen-depended transcription in nucleus [10,12]. Except its oestrogen potential, ZEA is hepatotoxic and immunotoxic [10]. However, since there is no evidence on DON and ZEA carcinogenicity the International Agency for Research on Cancer (IARC) has categorised DON and ZEA in group 3, not classifiable regarding their carcinogenicity to humans [13]. Nevertheless, in order to protect animal and human health the European Commission established regulatory guidance values and/or maximum levels for DON and ZEA in various commodities [14].

At cellular level, DON inhibits protein synthesis, activates mitogen-activated protein kinases (MAPKs) and induces apoptosis [8,9,15]. It is demonstrated that DON affects cell cycle and induces over-production of reactive oxygen species (ROS), resulting in oxidative damage of cell’s macromolecules [16]. Several studies confirmed genotoxic potential of DON [15,17,18]. Similarly, it is demonstrated that ZEA inhibits protein and DNA synthesis and induces oxidative stress causing lipid peroxidation (LPO) and oxidative DNA damage [10]. Moreover, ZEA reduces cell proliferation and increases apoptosis [11,12]. However, mechanisms underlying DON and ZEA toxicity are still not completely understood.

Due to frequent co-occurrence of DON and ZEA in various agricultural commodities and the resulting co-exposure in humans, this study aimed to explore the cytotoxicity and underlying mechanism of toxicity of DON and ZEA as single compounds or in combinations in human hepatocellular carcinoma (HepG2) cells. HepG2 cells were selected since both mycotoxins are metabolised in liver and are proven hepatotoxins [8,19]. HepG2 cells are liver cells of human origin that have retained the metabolic enzymes, hence they have the ability to imitate the conditions present in the organism that are particularly important for compounds needing metabolic transformation to exert their full toxicological activity [20,21,22]. In the first step of the study, we investigated the cytotoxicity of DON and ZEA and their mixtures using HepG2 cells. Afterwards, the cells were exposed to DON, ZEA, and their mixtures, to study genotoxicity, their impact on cell cycle, and cell proliferation. Genotoxicity was evaluated by the alkaline comet assay and γ-H2AX assay, while the impact of DON and ZEA and their mixtures on cell cycle and cell proliferation was assessed using flow cytometry.

## 2. Results

### 2.1. Cytotoxicity of DON, ZEA and Their Mixtures to HepG2 Cells

**Figure 1:**
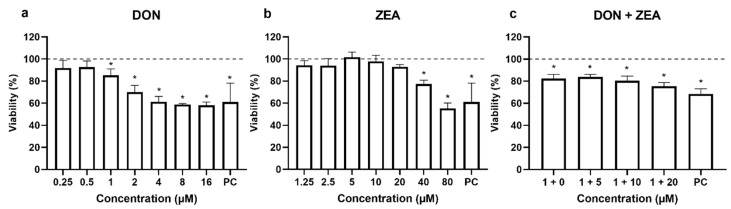
Viability of HepG2 cells (assessed by the MTS assay) after 24 h exposure to (**a**) deoxynivalenol (DON), (**b**) zearalenone (ZEA) or (**c**) their combinations (DON + ZEA). PC—positive control (30 μg/mL etoposide). * different from control, *p* < 0.05 (ANOVA; Dunnett’s multiple comparison test).

From the results, it can be observed that DON was more toxic to HepG2 cells compared to ZEA (Figure 1a,b). DON (1 μM) reduced HepG2 viability for 14.6% in average compared to solvent control (Figure 1a). Higher DON concentrations (2, 4, 8 and 16 μM) reduced cell viability by 29.8, 38.6, 41.1 and 42.0% in average, respectively (*p* < 0.05). However, ZEA only at the two highest concentrations applied in the study significantly lowered cell viability (40 μM for 22.6% and 80 μM for and 44.8%, Figure 1b).

The HepG2 cells exposed to DON (at the lowest cytotoxic concentration: 1 µM) and ZEA (at non-cytotoxic concentrations: 5, 10 and 20 µM) mixture significantly reduced HepG2 viability compared to solvent control (Figure 1c). Since ZEA at concentrations 5, 10 and 20 µM was not cytotoxic to HepG2 cells, it can be observed that the combination of both mycotoxins potentiated the effects of single compounds particularly at higher ZEA concentrations.

### 2.2. Genotoxic Activity of DON, ZEA and Their Mixtures in HepG2 Cells

**Figure 2:**
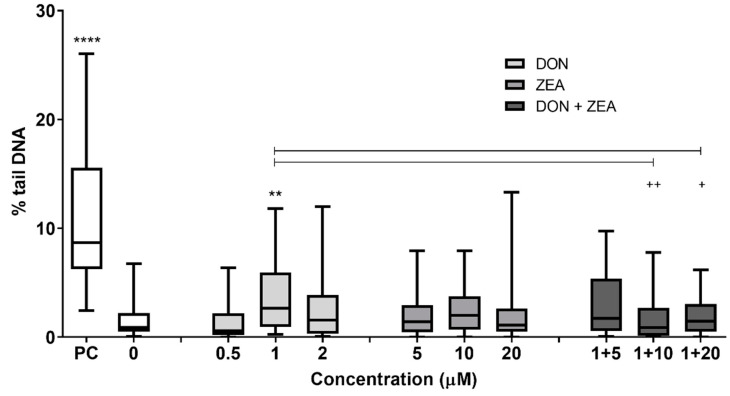
DNA strand breaks in HepG2 cells (assessed by the alkaline comet assay) after 24 h exposure to deoxynivalenol (DON), zearalenone (ZEA) or their combinations (DON+ZEA). 0—solvent control (0.1% DMSO), PC—positive control (30 μM B[a]P). ** different from control, *p* < 0.01; **** different from control, *p* < 0.001; ^+^ different from DON (1 µM), *p* < 0.05; ^++^ different from DON (1 µM), *p* < 0.01 (ANOVA; Scheffe’s multiple comparison test).

To assess genotoxic activity of DON, ZEA and their mixtures, the alkaline comet and γ-H2AX assays were applied. The alkaline comet assay is used to detect several DNA lesions such as DNA single-strand breaks (SSBs), alkali labile sites (ALS), SSBs formed during incomplete excision repair, apyrimidinic/apurinic sites (AP), as well as double-strand breaks (DSBs) [23,24,25]. A statistically significant higher DNA damage in HepG2 cells was only observed after exposure to DON at 1 µM, while at 2 µM, although elevated, it did not statistically differ compared to the control group (Figure 2). On the contrary, ZEA did not induce DNA strand break formation at tested concentrations (up to 20 µM). When testing the mixture, increased, however, non-significant DNA damage was detected at the combination of 1 µM DON and 5 µM ZEA compared to control (Figure 2). When comparing the combinations with DON (1 µM), significantly less DNA damage was observed at higher concentrations of ZEA (10 and 20 µM) compared to DON alone at 1 µM, suggesting an antagonistic effect between DON and ZEA (Figure 2).

**Figure 3:**
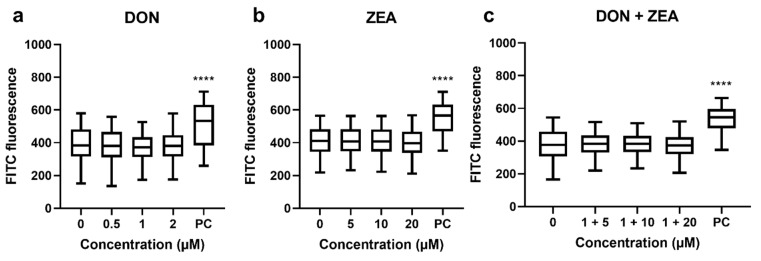
DNA double-strand breaks in HepG2 cells (assessed by the γ-H2AX formation using flow cytometry) after 24 h exposure to (**a**) deoxynivalenol (DON), (**b**) zearalenone (ZEA) or (**c**) their combinations (DON + ZEA). 0—solvent control (0.1% DMSO), PC—positive control (1 μg/mL etoposide). **** different from control, *p* < 0.001 (Mixed Effects Models (nlme) by REML).

Double-strand DNA breaks are the most harmful form of DNA damage, contributing to genomic instability, and if left unrepaired promote carcinogenic transformation of the cell [26]. γ-H2AX is a phosphorylated form of the H2AX histone, and it is used as a very sensitive biomarker of DNA DSBs. H2AX is phosphorylated on its 139th serine residue as a reaction to formation of DSB on DNA molecule, creating foci next to the break’s sites [27]. The whole phosphorylation process is fast, abundant and associates well with the number of DSBs, and therefore it is considered a highly sensitive biomarker of DNA DSBs induction [24,28]. In our case, DNA DSBs in HepG2 cells exposed to DON and ZEA or their mixtures were estimated by detection of γ-H2AX signals using flow cytometer. The results revealed that DON, ZEA and their combinations did not induce DNA DSBs at any concentration applied (Figure 3).

### 2.3. Effect of DON, ZEA and Their Mixtures on Cell Cycle Progression of HepG2 Cells

**Figure 4:**
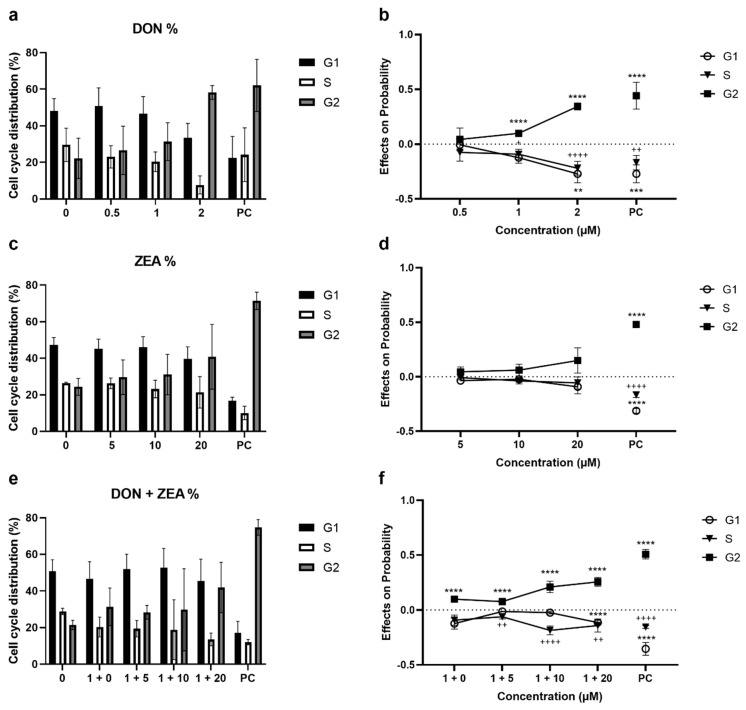
Distribution of HepG2 cells across the cell cycle phases (assessed by flow cytometry) and calculated effects of predicted probabilities with 95% Cls after 24 h exposure to (**a**,**b**) deoxynivalenol (DON), (**c**,**d**) zearalenone (ZEA) or (**e**,**f**) their combinations (DON + ZEA). 0—solvent control (0.1% DMSO), PC—positive control (1 μg/mL etoposide). ** *p* < 0.01 G1 different from control; *** *p* < 0.001 G1 different from control; ^+^ *p* < 0.05, ^++^ *p* < 0.01 S different from control; ^++++^ *p* < 0.001 S different from control; **** *p* < 0.001 G2 different from control (multinomial logistic regression).

We performed a flow cytometric cell cycle analysis of HepG2 cells exposed to DON and ZEA as single mycotoxins or in combination after staining DNA with Hoechst 33258. 24 h exposure to DON as single compound increased the G2-phase cells percentage in a concentration-dependent manner (Figure 4a,b). The percentage of G2-phase cells at 2 μM DON was approximately 58%, which was significantly higher than in the control (the percentage of cells in G2-phase in control was approximately 22%). In parallel, the percentage of G1-phase cells and S-phase cells decreased; the percentage of S-phase cells at 2 μM DON was approximately 8% that was significantly different from control (approximately 30%) (Figure 4a). A multinomial logistic regression analysis of results demonstrated that DON has the ability to arrest cells in cell cycle phase G2 (Figure 4b). Moreover, the predicted probability suggested that the exposure to DON (2 µM) significantly increased the number of cells in G2-phase (by 0.34% points) and simultaneously decreased the number of cells in the phases G1 and S (by −0.12 and −0.22% points, respectively), confirming the cell cycle arrest in the G2-phase.

A similar trend, the increase in the G2 phase cells, and a decrease in the G1 and S phases cells, was observed in HepG2 cells after 24 h exposure to ZEA, but the changes were not significant compared to control (Figure 4c,d).

The co-exposure of HepG2 cells to DON (1 µM) and ZEA (5, 10, and 20 µM) for 24 h significantly changed the distribution of HepG2 cells across the phases of cell cycle compared to control (Figure 4e,f). The effect of DON at 1 μM was more pronounced in the combination with ZEA when compared to DON alone at 1 μM and ZEA concentrations at 10 µM and 20 μM that alone did not affect cell distribution between cell cycle phases.

### 2.4. Effect of DON, ZEA and Their Mixtures on the Proliferation of HepG2 Cells

**Figure 5:**
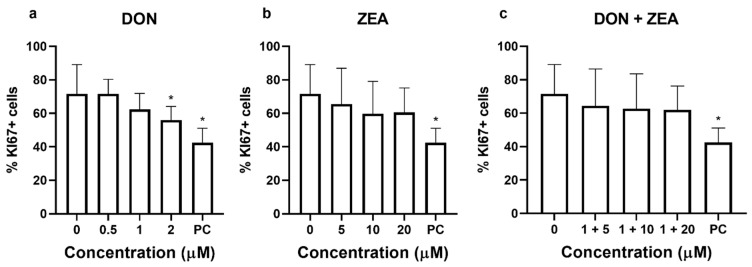
Proliferation of HepG2 cells (assessed by the level of Ki67 protein using flow cytometry) after 24 h exposure to (**a**) deoxynivalenol (DON), (**b**) zearalenone (ZEA) or (**c**) their combinations (DON + ZEA). 0—solvent control (0.1% DMSO), PC—positive control (1 μg/mL etoposide). * different from control, *p* < 0.05 (ANOVA; Dunnett’s multiple comparison test).

HepG2 cell proliferation was assessed by monitoring the proliferation-related protein Ki67 by flow cytometry. Ki67 protein is absent in the inactive phase (G0 phase) of cell cycle, while it appears in the active phases (G1—M phases) and is therefore commonly used as a biomarker of cell proliferation [29]. The 24 h exposure of HepG2 cells to DON caused a concentration-dependent reduction in the percentage of proliferating cells. At the highest DON concentration (2 µM) significant reduction is recorded that accounted approximately 55% of all cells compared to approximately 70% of proliferating control cells (Figure 5a). ZEA at tested concentrations showed a trend of decrease in Ki67 positive cells, but results did not differ statistically from the control (Figure 5b).

The combination of DON (1 µM) and ZEA (5, 10, and 20 µM) reduced the percentage of proliferating cells after 24 h exposure; however, the reduction did not differ statistically from the control. The percentage of proliferating cells after DON and ZEA co-exposure at 1 µM and 20 µM, respectively, was approximately 60% of all cells (compared to approximately 70% of proliferating control cells) (Figure 5c). No difference between the exposure of single compounds and their combinations at respective concentrations was observed.

## 3. Discussion

*Fusarium* mycotoxins, DON and ZEA, commonly co-contaminate feed and food, making human co-exposure to them unavoidable as confirmed by their co-occurrence in human urine samples [1,5,6]. From feed and food safety perspective, it is important to investigate interactions between co-occurring mycotoxins since their toxicity can differ from that of single mycotoxin [30,31,32,33]. As there is insufficient information available in the literature on the interactions between DON and ZEA with respect to their toxicity and mechanism of action, we therefore investigated the impact of DON and ZEA as single mycotoxins and their possible interactions on HepG2 cell viability, their genotoxic activity, and their impact on cell proliferation, and cell cycle. Based on the available literature, this is the first study exploring their combined effects on the cell proliferation and cell cycle.

The results indicated that both mycotoxins were cytotoxic to HepG2 cells. Previously, Juan-García et al. [34], reported IC_50_ value for DON on HepG2 cells after 24 h exposure to be 10.15 ± 0.41 μM, while after 48 h exposure the value was 0.23 ppm (approximately 0.76 μM) [35]. Additionally, Mayer et al. [8] reported that 24 h exposure to DON at concentration 0.9 μM reduced HepG2 cell viability to around 70%. In our study, DON at 1 μM decreased cell viability to approximately 85%. In contrast, in our study ZEA at 40 and 80 μM, decreased HepG2 cell viability to approximately 77 and 55%, respectively. The lower cytotoxic activity of ZEA on HepG2 cells was observed previously, with an IC_50_ of 41.28 μM after 24 h exposure and an IC_50_ of 6.45 ppm (approximately 20.4 μM) after 48 h exposure [10,33]. The results from our study on HepG2 cells are in accordance with the previously published data indicating that DON is more potent than ZEA. In addition, DON was more toxic than ZEA to murine leukaemia virus-induced tumour cells (RAW 264.7) [33], swine jejunal epithelial cells (IPEC-J2) [30], and to porcine lymphocytes [32]. However, Kouadio et al. [35] found that ZEA was more toxic to Caco-2 cells than DON, and IC_50_ after 72 h exposure of the cells to ZEA and DON were 15 μM and 21.5 μM, respectively.

Based on our cytotoxicity results as well as literature data showing that DON is more potent than ZEA to HepG2 cells [33], and since DON is detected in higher concentrations in commodities than ZEA [1], for studying combined effects we selected one DON concentration with slight cytotoxic activity (reduction of HepG2 cell viability for 15% in average) and three non-cytotoxic ZEA concentrations. The results showed that cytotoxic effects of DON and ZEA mixtures were more pronounced than effects induced by single compounds suggesting potentiating effects of both mycotoxins. Similarly, Zhou et al. [33] reported that in HepG2 and RAW 264.7 cells 48 h co-exposure to DON and ZEA at IC_50_ concentrations enhanced cytotoxic activity of individual toxins and the isobolagram analysis of interaction revealed their synergistic effect. However, at IC_25_ concentrations for RAW 264.7 cells additive effect was reported, suggesting that the type of combined effect depends on the concentration applied [33]. Wan et al. [30] in IPEC-J2 cells observed that DON and ZEA combination at non-cytotoxic concentrations (0.5 μM DON combined with 10 μM ZEA, 48 h) was cytotoxic. In an earlier study on Caco-2 cells, the 72 h co-exposure to DON and ZEA (10 μM + 10 μM or 20 μM + 20 μM) led to higher reduction of cell viability than exposure to single mycotoxin and the interaction was nearly additive [35]. On the contrary, 24 h co-exposure of porcine lymphocytes to DON and ZEA in low, non-cytotoxic concentrations (0.07 μg/mL DON combined with 5 μg/mL ZEA, corresponding to approximately 0.2 µM DON and 16.7 µM ZEA) resulted in no interaction, while after 48 h exposure to the same concentrations an antagonistic effect was recorded [32]. Taken together, those results show that the observed interactions between DON and ZEA vary from synergistic, additive and even antagonistic effects, and are clearly dependent on cell type, mycotoxin concentration and time of exposure. Wan et al. [30] observed in IPEC-J2 cells that co-exposure to ZEA and DON at non-cytotoxic concentrations resulted in cytotoxic effect. Our results support these finding since co-exposure to DON at concentration of very low toxicity and ZEA at non-cytotoxic concentrations resulted in higher toxicity. Thus, mixtures of mycotoxins in non-cytotoxic concentrations can have toxic effect. Such results are important since tested concentrations of DON and ZEA are actually relevant for the environment and thus human exposure [1,35,36,37]. Based on surveys conducted in EU countries, exposure estimates of dietary intake in children (the most exposed population group) for DON are up to 1.92 µg/kg b.w./day, and for ZEA 1.51 µg/kg b.w./day [38].

Data on DON and ZEA genotoxicity are scarce and inconclusive. Several studies reported that DON has genotoxic potential. In HepG2 cells, DON at 3.75 μM after 1 h exposure induced a dose-dependent increase in tail intensity using the comet assay, while at higher concentration (60 μM) and longer exposure (3 h) it induced oxidative DNA damage (detected as 8-OHdG) [39]. Moreover, in Caco-2 cells, DON at concentrations as low as 0.01 µM and 24 h exposure induced DNA damage [40]. Furthermore, in immortalised human keratinocyte HaCaT cells, DON at 1.68 μM after 6 h exposure caused oxidative DNA damage (detected as 8-OHdG), and after 12 h DSBs detected an increased level of γ-H2AX protein [41]. DSBs (quantified as increase of γ-H2AX) were also detected in rat intestinal epithelial IEC-6 cells after exposure to DON (12.5 μM, 8 h) [15]. On the other hand, after exposure of HepG2 cells to DON (2.4 and 4.8 μM, 48 h), Juan-García et al. [34] observed lower frequency of micronuclei compared to controls. Similarly, DON at cytotoxic concentration (up to 35 μM, 24 h) failed to induce micronuclei and formation of DNA strand breaks in human lymphoblastoid TK6 and human hepatoma HepaRG cell lines [42].

Genotoxic activity of ZEA (10 μM) was reported in transformed human primary embryonic kidney HEK293 cells, where primary DNA damage was induced after 2 h exposure [43] and in porcine granulosa cells, where 24 h exposure led to the formation of DSBs [11] detected by the comet and γ-H2AX assays, respectively.

Combined exposure of porcine lymphocytes to DON and ZEA at lower (0.07 μg/mL DON combined with 5 μg/mL ZEA, 24 h) and at higher (0.21 μg/mL DON combined with 10 μg/mL ZEA, 24 h) concentrations did not increase the tail intensity compared to single mycotoxin and control [32]. Similarly, DNA damage (assessed by the comet assay) was not detected in blood cells isolated from male Wistar rats treated with DON and ZEA as single and combined treatment (55 μg DON/kg b.w./day or 42.5 μg ZEA/kg b.w./day or 55 μg DON/kg b.w./day combined with 42.5 μg ZEA/kg b.w./day for 5 days, *i.p.*) [44]. In the present study, only DON at 1 µM significantly increased % tail DNA in HepG2 cells, while ZEA at applied concentrations had no impact on the formation of DNA strand breaks. The combination of DON (1 µM) with ZEA at higher concentrations (10 and 20 µM) reduced % tail DNA compared to 1 µM DON, indicating an antagonistic effect between DON and ZEA. On the contrary, DSBs were not detected in HepG2 cells after exposure to single mycotoxin or to their combination, as determined by γ-H2AX formation. Taken together, this indicates that genotoxic activity of DON, ZEA and their combinations depends on cell type, dose and length of exposure.

We also investigated the impact of DON, ZEA and their mixtures on the cell cycle and proliferation. According to the results, DON arrested HepG2 cells in the G2 phase dose dependently. The arrest of cell cycle in G1 or G2 phase is associated with the DNA damage repair. Additionally, cells can exit cell cycle in order to provide necessary time to re-establish cellular homeostasis or to coordinate the apoptosis initiation triggered by the external stress [36]. Previously, Juan-García et al. [34] reported that DON (0.6 and 1.2 μM; 48 h) increases the percentage of G2/M phase cells, while at higher concentration (4.8 μM) it increases the percentage of cells in Sub G0 and decreases in G2/M phase. Further, Yuan et al. [45] observed higher number of HepG2 cells in the G2/M phase after 6 h of exposure to 2 and 4 μg/mL of DON (corresponding to 6.6 and 13.2 μM) and correlated arrest of cell cycle at G2/M phase with the induction of p21 through ERG1 gene that was activated through stress-responsive transcription factor ATF3. Yang et al. [36] demonstrated that in human intestine-407 cells and intestinal epithelial HCT-116 cells, DON at 0.5–1 μg/L (corresponding to 1.6–3.3 µM) after 48 h arrested the cell cycle in G2/M phase by activating the p21, probably through ERK1/2 MAP kinase cascade. We further showed that the accumulation of HepG2 cells in G2 phase after exposure to DON was accompanied by the inhibition of cell proliferation that was assessed by measuring the Ki67 protein level. From the literature, it is known that DON can cause ribotoxic stress and block protein synthesis [45]; therefore, the observed decrease in cell proliferation may be related to the protein synthesis inhibition. In a study by Li et al. [46] on three different cell lines (Caco-2, HepG2 and Madin-Darby canine kidney (MDCK) cells), DON cytotoxicity was detected by MTT assay, but no LDH leakage was reported, thus the authors concluded that the cytotoxic effect of DON is not related to a disruption of membrane integrity. Similarly, reduction in cell viability after DON exposure observed in our study is unlikely due to a direct cytotoxic effect of DON but is rather related to arrest of cell cycle in the G2 phase and the inhibition of cell proliferation.

Moreover, for ZEA, the literature data show that it affects the cell cycle. In mouse Sertoli TM4 cells, a 24 h exposure to ZEA (10 µM) arrested cell cycle in G2/M phase and inhibited cell proliferation, with an approximately 15% decrease in viability [12]. Furthermore, cell arrest in the G2 phase was also reported in porcine granulosa cells after 24 h exposure to 30 µM ZEA [11]. Based on our results, ZEA showed non-significant arrest of HepG2 cells in G2 phase and showed a trend of reduced cell proliferation that was, however, non-significant. It should be noted that applied ZEA concentrations were not cytotoxic, as determined by the MTS assay. To the best of our knowledge, our study is the first to investigate the combined effects of DON and ZEA on the cell cycle and proliferation of HepG2 cells. According to the obtained results, the tested combination had similar effect as DON at applied condition, although a slight rise in accumulated G2 phase cells was observed at higher ZEA concentrations.

## 4. Materials and Methods

### 4.1. Chemicals and Preparation of Mycotoxins’ Standard Solution

Deoxynivalenol (DON, CAS-No. 51481-10-8; purity 98%), zearalenone (ZEA, CAS-No. 17924-92-4; purity 99%), dimethyl sulfoxide (DMSO), ethidium bromide (EtBr), benzo(a)pyrene (B[a]P), ethylenediaminetetraacetic acid (EDTA), minimum essential medium eagle (MEME-M2414) and its supplements were procured from Sigma (St. Louis, MO, USA). Etoposide (ET) was obtained from Santa Cruz Biotechnology (St. Cruz, CA, USA). Triton X-100 and foetal bovine serum (FBS) were purchased from Fisher Scientific (Branchburg, NJ, USA) and Tris was provided from Merck (Darmstadt, Germany). Antibodies and controls (Anti-Ki-67-PE-Vio770 (130-120-559), REA Control (I)-PE (130-104-613), Anti-H2AX pS139-FITC (130-118-478), and REA Control (I)-FITC (131-104-611)) were provided from Miltenyi Biotec (Bergisch Gladbach, Germany). Phosphate buffered saline (PBS) and ethanol were purchased from PAA Laboratories (Dartmouth, MA, USA), while low melting point agarose (LMP), Hoechst 33258 dye, and normal melting point agarose (NMP) were obtained from Invitrogen (Waltham, MA, USA) and the CellTiter 96^®^ AQueous cell proliferation assay (3-(4,5-dimethylthiazol-2-yl)-2,5-diphenyltetrazolium bromide; MTS) was purchased from Promega (Madison, WI, USA).

Standard stock solutions: 6 mM of DON and 20 mM of ZEA were prepared in DMSO and kept at −20 °C, and dilutions for cell treatment were prepared in cell medium each time fresh before treatment. The final concentration of DMSO in mycotoxin solutions for cell treatment did not exceed 0.1% and was adjusted to 0.1% in all concentrations.

### 4.2. Cell Culture

HepG2 cell line was obtained from the American Type Culture Collection ((ATCC), Manassas, VA, USA). The cells were grown in MEME medium supplemented with 2.2 g/L NaHCO_3_, 2 mM L-glutamine, 1% NEAA, 1 mM sodium pyruvate, 10% FBS, and 100 IU/mL streptomycin/penicillin. The cells were kept in a moisturised atmosphere with 5% CO_2_ at 37 °C.

### 4.3. Cytotoxicity—The MTS Assay

Cytotoxicity of DON, ZEA and their combinations was assessed by the tetrazolium-based (MTS) assay following the producer’s guides (Promega) with minor changes [47]. HepG2 cells were seeded into 96-well plate. After 24 h, cells were exposed to DON (0.25, 0.5, 1, 2, 4, 8 and 16 μM), ZEA (1.25, 2.5, 5, 10, 20, 40 and 80 μM) or to DON and ZEA (at concentrations: 1 μM DON + 5 μM ZEA, 1 μM DON + 10 μM ZEA, 1 μM DON + 20 μM ZEA) for 24 h. At the end of treatment period, we added 20% freshly prepared mixture of PMS:MTS solution (1:20) to each well and incubated for further 3 h. After the incubation period, absorbance was read at 490 nm on microplate reader (Sinergy MX, BioTek, Winooski, VT, USA). The experiment was conducted as five replicates per treatment point and repeated in three independent biological replicates. For each experiment we included negative control (cell medium), mycotoxin solvent control (cells exposed to 0.1% DMSO) and positive control (30 μg/mL ET).

The difference in cell viability among treated groups and solvent control group was analysed by the one-way analysis of variance (ANOVA) and Dunnett’s multiple comparison test with the program GraphPad Prism V8 (GraphPad Software, San Diego, CA, USA). The level of statistical significance was set at *p* < 0.05.

### 4.4. DNA Strand Breaks

The alkaline comet assay was performed according to Collins et al. [48] following recommendations by Møller et al. [49]. HepG2 cells at density 80,000 cells/well were seeded onto 12-well plates. After 24 h, the cells were exposed to ZEA (5, 10 and 20 μM), DON (0.5, 1 and 2 μM), or to the mixture of DON and ZEA (1 μM DON + 5 μM ZEA, 1 μM DON + 10 μM ZEA and 1 μM DON + 20 μM ZEA) for 24 h. In all experiments, we included negative (cell medium), solvent (0.1% DMSO) and positive controls (30 µM B[a]P). After 24 h of exposure, 30 μL of cell suspension was added to 70 μL 1% LMP agarose and placed onto 1% NMP agarose-precoated fully frosted slides. The slides were placed into lysis solution (1% Triton X-100, 100 mM EDTA, 2.5 M NaCl, 10 mM Tris, pH 10) at 4 °C for 1 h, following placement of the slides in electrophoresis buffer (300 mM NaOH, 1 mM EDTA, pH 13) at 4 °C for 20 min to allow DNA unwinding, and further 20 min of electrophoresis at 25 V (300 mA). Afterwards, the slides were washed with 0.4 M Tris buffer (pH 7.5) for 15 min. Prior to analysis, we stained the nuclei with EtBr (10 μg/mL) and analysed them using an Orthoplan epifluorescence microscope (Leitz, Germany). To assess the level of DNA damage, we chose the percent of tail DNA (% of tail DNA). Per experimental point, a total of 100 randomly captured nuclei was analysed using the Comet Assay II (Perceptive Instruments Ltd., Haverhill, UK) software. Experiments were carried out in duplicate and repeated as three independent biological replicates.

For the comet assay results, breakdown and one-way ANOVA were used to analyse the differences between the treatment groups and solvent control group within each experiment. Scheffé’s test was used for multiple comparisons versus the solvent control. The analysis was performed using STATISTICA 13.2 (Dell Inc., Round Rock, TX, USA). The level of statistical significance was set at *p* < 0.05.

### 4.5. Simultaneous Determination of the Gamma-H2AX Positive Cells, Cell Proliferation and Cell Cycle by Flow Cytometry

The analysis of the γ-H2AX positive cells, cell proliferation and cell cycle was carried out using a flow cytometer on the fixed single-cell suspension after the 24 h exposure to DON (0.5, 1 and 2 μM), ZEA (5, 10 and 20 μM) or the mixture of DON and ZEA (1 μM DON + 5 μM ZEA, 1 μM DON + 10 μM ZEA, 1 μM DON + 20 μM ZEA). In experiments, negative control (cell medium), solvent control (0.1% DMSO) and positive control (1 μg/mL ET) were included. Cells were first fixed in 70% ethanol, washed in cold PBS and then labelled with anti-KI67-PE Vio770 and anti-H2AX pS139-FITC (diluted 1:50 in 1% BSA) for 30 min, washed again with PBS, and afterwards incubated with Hoechst 33258 dye (diluted 1:500 in 0.1% Triton X-100) for 20 min as reported by Hercog et al. [48] and Štampar et al. [50]. MACSQuant^®^ Analyzer 10 (Miltenyi Biotech, Bergisch Gladbach, Germany) flow cytometer using FlowJo v10 software (Becton, Dickinson and Company, Franklin Lakes, NJ, USA) was used for the analysis. FITC intensity correlating to DSBs was detected in the B1-A channel, PE Vio770 intensity correlating to KI67 positive cells was detected in the B3-A channel and Hoechst fluorescence correlating to cell cycle was detected in the V1-A channel as reported by Hercog et al. [48] and Ujvarosi et al. [51]. To exclude unspecific binding, we used internal REA controls. For each experimental point, we recorded 2.5 × 10^4^ single cells, and repeated the experiments in three independent biological repetitions.

Statistical significance of H2AX positive cells between treated groups and control was determined using exported .csv values in the R software with the Mixed Effects Models (nlme) package by REML as described in Ramaiahgari et al. [52]. The statistical analysis of Ki67 positive cells was analysed by the one-way ANOVA with the posthoc multiple comparisons Dunnett’s test using GraphPad Prism V8 (GraphPad Software), while the cell cycle distribution of the solvent control and treated samples was conducted by the multinomial logistic regression, and further post estimation tests in Stata 15 (StataCorp LLC, College Station, TX, USA) [53]. The level of statistical significance was set at *p* < 0.05.

## 5. Conclusions

Taken together, our study showed that DON induced cytotoxic effects in HepG2 cells at lower concentrations compared to ZEA. The combined cytotoxic effects were more pronounced than those induced by individual compounds, in particular for DON, suggesting that the combined effects of both mycotoxins are potentiated. Using the comet assay, we detected DNA damage only for DON at 1 µM, while the combination of DON and higher ZEA concentrations reduced DNA strand break formation compared to 1 µM DON indicating antagonistic effects. However, none of the mycotoxins or their combinations induced DSBs. Both mycotoxins and their combinations dose dependently arrested the cell cycle in G2 phase, with DON being more toxic. These effects were observed at low, environmentally relevant concentrations, highlighting the problem of potential human co-exposure to multiple mycotoxins in everyday life and their possible interactions, as well as an urgent need for setting new regulations/risk assessments considering their interactions. Currently government/regulatory agencies base their recommendations on toxicological data of single mycotoxin. However, mycotoxins often co-occur in nature and their interactions should be taken into account when predicting risk assessment and setting new recommendations.

## Data Availability

The data presented in this study are available on request from the authors.

## References

[1] Gruber-Dorninger C., Jenkins T., Schatzmayr G. (2019). Global Mycotoxin Occurrence in Feed: A Ten-Year Survey. Toxins.

[2] Pleadin J., Sokolović M., Perši N., Zadravec M., Jaki V., Vulić A. (2012). Contamination of maize with deoxynivalenol and zearalenone in Croatia. Food Control.

[3] Bertuzzi T., Rastelli S., Mulazzi A., Donadini G., Pietri A. (2011). Mycotoxin occurrence in beer produced in several European countries. Food Control.

[4] Peters J., Van Dam R., Van Doorn R., Katerere D., Berthiller F., Haasnoot W., Nielen M.W.F. (2017). Mycotoxin profiling of 1000 beer samples with a special focus on craft beer. PLoS ONE.

[5] Heyndrickx E., Sioen I., Huybrechts B., Callebaut A., De Henauw S., De Saeger S. (2015). Human biomonitoring of multiple mycotoxins in the Belgian population: Results of the BIOMYCO study. Environ. Int..

[6] Solfrizzo M., Gambacorta L., Visconti A. (2014). Assessment of Multi-Mycotoxin Exposure in Southern Italy by Urinary Multi-Biomarker Determination. Toxins.

[7] Turner P.C., Rothwell J., White K.L.M., Gong Y.Y., Cade J., Wild C.P. (2008). Urinary Deoxynivalenol Is Correlated with Cereal Intake in Individuals from the United Kingdom. Environ. Health Perspect..

[8] Mayer E., Novak B., Springler A., Schwartz-Zimmermann H.E., Nagl V., Reisinger N., Hessenberger S., Schatzmayr G. (2017). Effects of deoxynivalenol (DON) and its microbial biotransformation product deepoxy-deoxynivalenol (DOM-1) on a trout, pig, mouse, and human cell line. Mycotoxin Res..

[9] Pestka J.J., Smolinski A.T. (2005). Deoxynivalenol: Toxicology and Potential Effects on Humans. J. Toxicol. Environ. Health Part B.

[10] Li Y., Zhang B., He X., Cheng W.-H., Xu W., Luo Y., Liang R., Luo H., Huang K. (2014). Analysis of Individual and Combined Effects of Ochratoxin A and Zearalenone on HepG2 and KK-1 Cells with Mathematical Models. Toxins.

[11] Liu X.-L., Wu R.-Y., Sun X.-F., Cheng S.-F., Zhang R.-Q., Zhang T.-Y., Zhang X.-F., Zhao Y., Shen W., Li L. (2018). Mycotoxin zearalenone exposure impairs genomic stability of swine follicular granulosa cells in vitro. Int. J. Biol. Sci..

[12] Zheng W.-L., Wang B.-J., Wang L., Shan Y.-P., Zou H., Song R.-L., Wang T., Gu J.-H., Yuan Y., Liu X.-Z. (2018). ROS-Mediated Cell Cycle Arrest and Apoptosis Induced by Zearalenone in Mouse Sertoli Cells via ER Stress and the ATP/AMPK Pathway. Toxins.

[13] IARC (1993). Some Naturally Occurring Substances: Food Items and Constituents, Heterocyclic aromatic Amines and Mycotoxins.

[14] European Commission (2006). Commission Recommendation of 17 August 2006 on the presence of deoxynivalenol, zearalenone, ochratoxin A, T-2 and HT-2 and fumonisins in products intended for animal feeding. Off. J. Eur. Union.

[15] Payros D., Dobrindt U., Martin P., Secher T., Bracarense A.P.F.L., Boury M., Laffitte J., Pinton P., Oswald E., Oswald I.P. (2017). The Food Contaminant Deoxynivalenol Exacerbates the Genotoxicity of Gut Microbiota. Mbio.

[16] Lei Y., Guanghui Z., Xi W., Yingting W., Xialu L., Fangfang Y., Goldring M.B., Xiong G., Lammi M.J. (2017). Cellular responses to T-2 toxin and/or deoxynivalenol that induce cartilage damage are not specific to chondrocytes. Sci. Rep..

[17] Chen S.S., Li Y.-H., Lin M.-F. (2017). Chronic Exposure to the Fusarium Mycotoxin Deoxynivalenol: Impact on Performance, Immune Organ, and Intestinal Integrity of Slow-Growing Chickens. Toxins.

[18] Singh S., Banerjee S., Chattopadhyay P., Borthakur S.K., Veer V. (2015). Deoxynivalenol induces cytotoxicity and genotoxicity in animal primary cell culture. Toxicol. Mech. Methods.

[19] Lorenz N., Dänicke S., Edler L., Gottschalk C., Lassek E., Marko D., Rychlik M., Mally A. (2019). A critical evaluation of health risk assessment of modified mycotoxins with a special focus on zearalenone. Mycotoxin Res..

[20] Ehrlich V., Darroudi F., Uhl M., Steinkellner H., Zsivkovits M., Knasmueller S. (2002). Fumonisin B1 is genotoxic in human derived hepatoma (HepG2) cells. Mutagenesis.

[21] Knasmüller S., Mersch-Sundermann V., Kevekordes S., Darroudi F., Huber W.W., Hoelzl C., Bichler J., Majer B.J. (2004). Use of human-derived liver cell lines for the detection of environmental and dietary genotoxicants; current state of knowledge. Toxicology.

[22] Waldherr M., Mišík M., Ferk F., Tomc J., Žegura B., Filipič M., Mikulits W., Mai S., Haas O., Huber W.W. (2018). Use of HuH6 and other human-derived hepatoma lines for the detection of genotoxins: A new hope for laboratory animals?. Arch. Toxicol..

[23] Azqueta A., Ladeira C., Giovannelli L., Boutet-Robinet E., Bonassi S., Neri M., Gajski G., Duthie S., Del Bo’ C., Riso P. (2020). Application of the comet assay in human biomonitoring: An hCOMET perspective. Mutat. Res. Rev. Mutat. Res..

[24] Gerić M., Gajski G., Garaj-Vrhovac V. (2014). γ-H2AX as a biomarker for DNA double-strand breaks in ecotoxicology. Ecotoxicol. Environ. Saf..

[25] Žegura B., Filipič M., Yan Z., Caldwell G. (2004). Application of In Vitro Comet Assay for Genotoxicity Testing. Optimization in Drug Discovery, Methods in Pharmacology and Toxicology.

[26] Khanna K.K., Jackson S.P. (2001). DNA double-strand breaks: Signaling, repair and the cancer connection. Nat. Genet..

[27] Kopp B., Khoury L., Audebert M. (2019). Validation of the γH2AX biomarker for genotoxicity assessment: A review. Arch. Toxicol..

[28] Rogakou E.P., Pilch D.R., Orr A.H., Ivanova V.S., Bonner W.M. (1998). DNA Double-stranded Breaks Induce Histone H2AX Phosphorylation on Serine 139. J. Biol. Chem..

[29] Miller I., Min M., Yang C., Tian C., Gookin S., Carter D., Spencer S.L. (2018). Ki67 is a Graded Rather than a Binary Marker of Proliferation versus Quiescence. Cell Rep..

[30] Wan L.Y.M., Turner P.C., El-Nezami H. (2013). Individual and combined cytotoxic effects of Fusarium toxins (deoxynivalenol, nivalenol, zearalenone and fumonisins B1) on swine jejunal epithelial cells. Food Chem. Toxicol..

[31] Smith M.-C., Madec S., Coton E., Hymery N. (2016). Natural Co-Occurrence of Mycotoxins in Foods and Feeds and Their in vitro Combined Toxicological Effects. Toxins.

[32] Kachlek M., Szabó-Fodor J., Bodnár Z.B., Horvatovich K., Kovács M. (2017). Preliminary results on the interactive effects of deoxynivalenol, zearalenone and fumonisin B1 on porcine lymphocytes. Acta Vet. Hung..

[33] Zhou H., George S., Hay C., Lee J., Qian H., Sun X. (2017). Individual and combined effects of Aflatoxin B 1, Deoxynivalenol and Zearalenone on HepG2 and RAW 264.7 cell lines. Food Chem. Toxicol..

[34] Juan-García A., Taroncher M., Font G., Ruiz M.-J. (2018). Micronucleus induction and cell cycle alterations produced by deoxynivalenol and its acetylated derivatives in individual and combined exposure on HepG2 cells. Food Chem. Toxicol..

[35] Kouadio J.H., Dano S.D., Moukha S., Mobio T.A., Creppy E.E. (2007). Effects of combinations of Fusarium mycotoxins on the inhibition of macromolecular synthesis, malondialdehyde levels, DNA methylation and fragmentation, and viability in Caco-2 cells. Toxicon.

[36] Yang H., Chung D.H., Kim Y.B., Choi Y.H., Moon Y. (2008). Ribotoxic mycotoxin deoxynivalenol induces G2/M cell cycle arrest via p21Cip/WAF1 mRNA stabilization in human epithelial cells. Toxicology.

[37] Streit E., Schatzmayr G., Tassis P., Tzika E., Marin D., Taranu I., Tabuc C., Nicolau A., Aprodu I., Puel O. (2012). Current Situation of Mycotoxin Contamination and Co-occurrence in Animal Feed—Focus on Europe. Toxins.

[38] Marin S., Ramos A.J., Cano-Sancho G., Sanchis V. (2013). Mycotoxins: Occurrence, toxicology, and exposure assessment. Food Chem. Toxicol..

[39] Zhang X., Jiang L., Geng C., Cao J., Zhong L. (2009). The role of oxidative stress in deoxynivalenol-induced DNA damage in HepG2 cells. Toxicon.

[40] Bony S., Carcelen M., Olivier L., Devaux A. (2006). Genotoxicity assessment of deoxynivalenol in the Caco-2 cell line model using the Comet assay. Toxicol. Lett..

[41] Mishra S., Tewari P., Chaudhari B.P., Dwivedi P.D., Pandey H.P., Das M. (2016). Deoxynivalenol induced mouse skin tumor initiation: Elucidation of molecular mechanisms in human HaCaT keratinocytes. Int. J. Cancer.

[42] Takakura N., Nesslany F., Fessard V., Le Hegarat L. (2014). Absence of in vitro genotoxicity potential of the mycotoxin deoxynivalenol in bacteria and in human TK6 and HepaRG cell lines. Food Chem. Toxicol..

[43] Gao F., Jiang L.-P., Chen M., Geng C.-Y., Yang G., Ji F., Zhong L.-F., Liu X.-F. (2013). Genotoxic effects induced by zearalenone in a human embryonic kidney cell line. Mutat. Res. Toxicol. Environ. Mutagen..

[44] Szabó-Fodor J., Szabó A., Kócsó D., Marosi K., Bóta B., Kachlek M., Mézes M., Balogh K., Kövér G., Nagy I. (2019). Interaction between the three frequently co-occurring *Fusarium* mycotoxins in rats. J. Anim. Physiol. Anim. Nutr..

[45] Yuan L., Mu P., Huang B., Li H., Mu H., Deng Y. (2018). EGR1 is essential for deoxynivalenol-induced G2/M cell cycle arrest in HepG2 cells via the ATF3ΔZip2a/2b-EGR1-p21 pathway. Toxicol. Lett..

[46] Li X., Mu P., Wen J., Deng Y. (2017). Carrier-Mediated and Energy-Dependent Uptake and Efflux of Deoxynivalenol in Mammalian Cells. Sci. Rep..

[47] Hercog K., Maisanaba S., Filipič M., Sollner-Dolenc M., Kač L., Žegura B. (2019). Genotoxic activity of bisphenol A and its analogues bisphenol S, bisphenol F and bisphenol AF and their mixtures in human hepatocellular carcinoma (HepG2) cells. Sci. Total. Environ..

[48] Collins A., Møller P., Gajski G., Vodenková S., Abdulwahed A., Anderson D., Bankoglu E.E., Bonassi S., Boutet-Robinet E., Brunborg G. (2023). Measuring DNA modifications with the comet assay: A compendium of protocols. Nat. Protoc..

[49] Møller P., Azqueta A., Boutet-Robinet E., Koppen G., Bonassi S., Milić M., Gajski G., Costa S., Teixeira J.P., Pereira C.C. (2020). Minimum Information for Reporting on the Comet Assay (MIRCA): Recommendations for describing comet assay procedures and results. Nat. Protoc..

[50] Štampar M., Breznik B., Filipič M., Žegura B. (2020). Characterization of In Vitro 3D Cell Model Developed from Human Hepatocellular Carcinoma (HepG2) Cell Line. Cells.

[51] Ujvárosi A.Z., Hercog K., Riba M., Gonda S., Filipič M., Vasas G., Žegura B. (2020). The cyanobacterial oligopeptides microginins induce DNA damage in the human hepatocellular carcinoma (HepG2) cell line. Chemosphere.

[52] Ramaiahgari S.C., den Braver M.W., Herpers B., Terpstra V., Commandeur J.N.M., Van De Water B., Price L.S. (2014). A 3D in vitro model of differentiated HepG2 cell spheroids with improved liver-like properties for repeated dose high-throughput toxicity studies. Arch. Toxicol..

[53] Štampar M., Žabkar S., Filipič M., Žegura B. (2022). HepG2 spheroids as a biosensor-like cell-based system for (geno)toxicity assessment. Chemosphere.

